# Telehealth for management of chronic non-cancer pain and opioid use disorder in safety net primary care

**DOI:** 10.1186/s12913-023-09330-w

**Published:** 2023-04-01

**Authors:** Alexis Cooke, Stacy Castellanos, Celeste Enriquez, Pamela Olsen, Christine Miaskowski, Margot Kushel, Kelly Ray Knight

**Affiliations:** 1 Department of Community Health Systems, School of Nursing, University of California - San Francisco, 2 Koret Way, N505, San Francisco, CA 94143-0608 USA; 2 Department of Humanities and Social Sciences, School of Medicine, University of California - San Francisco, 490 Illinois Street, 7th Floor, San Francisco, CA 94143-0850 USA; 3 Center for Vulnerable Populations at Zuckerberg San Francisco General Hospital and Trauma Center, Department of Medicine, School of Medicine, University of California - San Francisco, UCSF Box 1339, San Francisco, CA 94143-0608 USA; 4 Department of Physiological Nursing, School of Nursing, University of California - San Francisco, 2 Koret Way, Rm 631, San Francisco, CA 94143-0608 USA; 5 Department of Humanities and Social Sciences, School of Medicine, University of California - San Francisco, 490 Illinois Street, 7th Floor, San Francisco, CA 94143-0608 USA

**Keywords:** Chronic non-cancer pain, Opioids, Pain management, Primary care, Qualitative, Telehealth, Telemedicine

## Abstract

**Background:**

The SARS-CoV-2 (COVID-19) pandemic increased use of telehealth for the management of opioid use disorder and chronic non-cancer pain in primary care safety net clinical systems. Significant barriers to telehealth exist, little is known about how these barriers impact urban safety net, primary care providers and their patients. The objective of this study was to qualitatively assess the benefits and challenges of telehealth for management of chronic non-cancer pain, opioid use disorder, and multi-morbidity in primary care, safety net clinical systems.

**Methods:**

We interviewed patients with chronic non-cancer pain and history of substance use (*n* = 22) and their primary care clinicians (*n* = 7) in the San Francisco Bay Area, March-July 2020. We recorded, transcribed, coded, and content analyzed interviews.

**Results:**

COVID-19 shelter-in-place orders contributed to increases in substance use and uncontrolled pain, and posed challenges for monitoring opioid safety and misuse through telehealth. None of the clinics used video visits due to low digital literacy/access. Benefits of telehealth included decreased patient burden and missed appointments and increased convenience and control of some chronic conditions (e.g., diabetes, hypertension). Telehealth challenges included loss of contact, greater miscommunication, and less comprehensive care interactions.

**Conclusions:**

This study is one of the first to examine telehealth use in urban safety net primary care patients with co-occurring chronic non-cancer pain and substance use. Decisions to continue or expand telehealth should consider patient burden, communication and technology challenges, pain control, opioid misuse, and medical complexity.

## Background

Opioid use disorder and chronic non-cancer pain are common in the United States (US), as is their co-occurrence [1–3]. Over 80 million Americans report chronic non-cancer pain, defined as non-malignant pain that lasts longer than three months, not associated with end of life [4, 5]. Approximately, 4.8 million adults in the US have a current or past opioid use disorder diagnosis [6]. The majority of chronic non-cancer pain management, and of opioid prescriptions, occurs in primary care settings rather than in pain specialty clinics [7]. In response to the excess morbidity and mortality associated with opioid prescribing, primary care clinics implemented multiple interventions to improve opioid use disorder and chronic non-cancer pain management [8]. These interventions include the mandated use of Prescription Drug Monitoring Programs (PDMP), systematic tapering of patients’ opioid prescriptions, as well as proactive efforts, such as improved provider training to assess and diagnose opioid use disorder, expanded access to non-opioid chronic non-cancer pain treatment modalities (e.g., acupuncture, physical therapy), and increased availability of buprenorphine to treat opioid use disorder [9, 10].

The onset of the SARS-CoV-2 (COVID-19) pandemic in the US created an urgent need for in-person care alternatives for outpatient primary care that could reduce patient and provider risks for COVID-19 infection and allow for the redeployment of clinical resources toward COVID-19-related patient assessment and critical care [11]. Since its inception, telehealth has expanded to include video calls, telephone calls, online algorithms and asynchronous monitoring (e.g., text messages), and remote monitoring (the use of wearable sensors and mobile diagnostic systems) [12]. However, significant barriers to telehealth exist including: limited insurance reimbursement, particularly with Medicare; patient and provider discomfort with telehealth technologies; and differential access to telecommunication [12]. Little is known about how these barriers impact urban safety net, primary care providers and their patients with co-occurring chronic non-cancer pain, opioid use disorder, and multiple morbidity.

At multiple institutions, the number of telehealth visits increased substantially following stay-at-home orders and other pandemic restrictions [13, 14]. For patients with a substance use disorder, traditional forms of patient monitoring (e.g., in person toxicology screens) and non-opioid chronic non-cancer pain treatment modalities were postponed or discontinued [15, 16]. While limited research has documented the successful transition of opioid use disorder treatment to telehealth, little is known about the impact of using telehealth on the management of co-occurring opioid use disorder and chronic non-cancer pain, especially in urban settings [17–19]. Safety net clinics are defined by the Institute of Medicine as those settings that “…offer care to patients regardless of their ability to pay for services, and [for which] a substantial share of their patients are uninsured, Medicaid, or other vulnerable patients” [7, 8]. We conducted a qualitative study of urban primary care, safety net providers and their patients with chronic non-cancer pain, a history of substance use, and multiple morbidities to understand the impacts of transitioning to telehealth.

## Methods

### Participation and recruitment

For this COVID-19 sub-study, we leveraged the procedures from an on-going study (“Examining the Consequences of Reductions in Opioid Prescribing” (ECROP) R01 DA043631) to interview active primary care provider and patient participants from four safety net primary care clinics across two counties in the San Francisco (SF) Bay Area, from March-July 2020. The ECROP parent study was a longitudinal, qualitative study that focused on examining the impact of opioid prescribing reductions in safety net primary care settings. ECROP study enrolled primary care providers with continuity practices and their patients who had a diagnosis of chronic non-cancer pain and a history of substance use.

### Data collection

We interviewed seven [7] primary care providers (providers) and conducted ethnographic check-ins and semi-structured interviews with 22 patients with chronic non-cancer pain. Provider interview guides focused on experiences transitioning to telehealth; benefits and challenges of telehealth; managing co-occurring chronic non-cancer pain and patients’ substance use; and patients’ mental health experiences during COVID-19. For each participating clinician, we recruited between one and four of their patients who had both chronic non-cancer pain and a history of past or current substance use (including illicit drugs and/or alcohol). Patient interviews focused on experiences transitioning to telehealth; daily pain severity and functionality; substance use and mental health experiences during COVID-19; and overall health and healthcare experiences. We conducted data collection activities over the telephone (with patients) or on Zoom™ (with providers) to reduce COVID-19 exposure for researchers and participants. Patient interviews were conducted over the telephone to promote participation, due to limited digital access among patients. We collected provider and patient demographic and health data at ECROP enrollment. The Institutional Review Board (IRB) of the University of California, San Francisco (UCSF) [Study# 16-21377] approved all study procedures and participants completed informed consent prior to participation in the study.

### Sample composition

All seven providers who participated in this sub-study were physicians. Providers’ age ranged from 35 to 55 years old and experience in primary care ranged from 5.5 to 28 years. A majority of providers identified as women (*n* = 6) and one provider identified as a man.

Self-reported patient age ranged from 42 to 83 years (60.5 ± 10.3). Twelve patients identified as women (54.5%), nine as men (40.9%), and one patient was genderqueer (4.5%). Patients reported racial or ethnic identity as: Black/African American (*n* = 10, 45.5%), White/European American (*n* = 8, 36.4%), Latina/Latino/Latinx (*n* = 3, 13.6%); and Asian/Asian American (*n* = 1, 4.5%). The majority of ECROP participants (66.6%) reported at least one chronic health condition (e.g., diabetes, hypertension, lung disease) in addition to substance use and chronic non-cancer pain.

### Analysis

All audio-recordings were transcribed verbatim and interviews and ethnographic check-ins were memo-ed immediately after data collection activities. To protect participant confidentiality, transcripts were de-identified and saved using an alphanumeric participant identification code. Data were stored using encrypted data storage only accessible to the study team. We modified the existing ECROP Study coding scheme to include COVID-related themes. To modify the codebook to include COVID-19-specific concepts, we reviewed transcripts and memos; conducted consensus meetings to summarize data; and developed an initial set of inductive codes for both provider and patient datasets. Using the initial codes, researchers jointly coded two transcripts from the provider and patient datasets. Then, we modified sets of codes to reflect additional emergent themes from the joint coding process. All transcripts were entered into Dedoose software for data management purposes [20]. For this analysis, we conducted a content analysis of Dedoose queries for “telehealth”, “substance use” and, “pain”, codes for both provider and patient datasets [21, 22].

## Results

### Provider and Patient Experiences of COVID-19 Response and Transitions to Telehealth

In response to SF Bay Area’s stay-at-home order issued on March 16, 2020, safety net primary care clinics reconfigured clinic operations within days and transitioned primary care to a telehealth format. Across clinics, providers identified concerns about patients being able to use the applications and device usage required to enable video-based telehealth appointments. Thus, clinics attempted few video visits with patients.

Patients described reductions in activity and movement that resulted in increases in uncontrolled pain while sheltering-in-place, that decreased functional status and mobility. Some patients linked increased pain severity to limited or inaccessible alternative pain management care (e.g., physical therapy, acupuncture, massage therapy). Patients shared how COVID-19 concerns and perceived isolation negatively impacted their mental health. One patient reported, “I would rate [my pain] the highest you can go…I’m in bed now and not even moving and it’s hurting…I can’t make it to the bathroom anyway.” Another patient stated: “[I am taking my pain medications] more…Because I’m not doing the exercises and the therapy, physical therapy.”

Providers reported that some patients sought opioids for pain relief in emergency departments; requested increased opioid doses or took more opioids than prescribed; and increased substance use in direct response to COVID-19-related anxiety and fears and disruptions in support systems. One provider stated: “[E]verybody is using more [substances]. I’ve had several patients relapse…I think again the combination of losing some of the other support structures, whether it was a job, or you know your regular [substance use treatment] meeting… then just increased fear and anxiety seems has driven people to either increase their use relapse.”

Providers described pausing opioid tapers that were in progress, and some supplemental opioid prescribing, due to reports of increased patient pain and disruptions in patient access to non-opioid pain treatments. One provider stated: “[I]t’s hard right because [providers] feel like even though…[opioids are] not warranted for long term use you could see why people are having more pain now. And they don’t really have access to all the…other modalities and treatments that are much more helpful like acupuncture, physical therapy, the regular exercise classes. There’s a couple [situations] where I kind of like [asked myself]: ‘Can we increase the [opioid] medicine just a little bit?’ A few people have like a short-term benefit…[I]t’s also psychological, [patients] feel better.”

Telehealth made the monitoring of opioid safety and misuse challenging, which contributed to providers’ concerns about opioid-associated injuries and hospitalizations. One provider reported: “[My patient] ended up in the hospital because she relied on a [carisoprodol] from a neighbor. And it made her dizzy and [she] felt very bad and she went to the emergency room for it. And I felt terrible…because I couldn’t evaluate her, I couldn’t really see what she was dealing with and [was] just using her verbal history [over the phone].”

### Benefits of Telehealth

Patients and providers described benefits from telehealth through telephone visits, including increased convenience and improved provider follow-up. Providers felt that telehealth improved patient attendance by lowering barriers to access (i.e., not having to travel to clinic), provided them insights regarding patients’ home life, and facilitated monitoring and follow-up of patients with chronic health conditions.

#### Reduced burden and increased convenience

Both patients and providers cited convenience as a major benefit of telehealth. Most patients relied on public transportation. Numerous bus routes and services to get to the clinics were suspended to reduce risk for COVID-19 infection, thus not having to travel for clinic visits reduced patient burden. For patients who required routine check-ups to refill medications or update their clinician about their general health status, telehealth visits offered a quick and efficient way to facilitate care. One patient commented: “[A]t least I don’t have to take the bus to get there you know or the train…And I don’t have to worry about being late.”

#### Improvements in follow-ups for chronic conditions and urgent health issues

Many patients reported perceived increases in providers’ responsiveness and access (e.g., having access to providers’ cell phone number). Providers suggested that telehealth allowed them to provide targeted care (both in-person and via telehealth) for more urgent issues. In-person availability was severely limited at study clinics, especially in the early months of the COVID-19 pandemic (March-June 2020). Conducting phone check-ins for non-urgent health issues freed providers to see patients with urgent needs (e.g., falls, acute injuries, severely uncontrolled pain) quickly in person. One provider stated: “[T]he show rates of phone visits are really high… [P]atient access was more immediate and if they had a fall obviously you can call them and you can call them throughout the day and you can follow up at some point in the day if you couldn’t reach them at that time.” Another provider remarked: “If the patient says, ‘I need to see you,’ I [say] ‘Come on in.’…Our availability has increased…really dramatically…my own person[al] availability for [in-person] critical care has almost exactly doubled [because of transitioning patients to telehealth].”

#### Insights into patients’ home environments

Providers reported that telehealth visits gave them greater insights into patients’ everyday lives because they could hear family members in the background. In addition, sometimes family members and caregivers joined telehealth visits, offering a more comprehensive understanding of factors impacting patients’ health. Providers noted that telehealth helped to facilitate medication checks, because patients could directly assess and report on their supply of medications from home. Prior to COVID-19, it was typical for patients to forget to bring in their medications to in-person clinical visits and recall could be poor. One provider reported: “I think taking one step to telehealth is it’s so helpful to take away this fictitious world of like I’ve seen a patient in an exam room….[Telehealth has] already taken down that whole construct in so many ways and allowed us to be real on the phone and say, ‘Okay, now I hear your child in the background, I hear your dog in the background.’ It’s a more real-life picture of what our patients are dealing with” Table 1.

**Table 1:** Benefits of telehealth

**Patients: convenience, reduced burden**
I think the calling is a good idea because you know for patients in my situation, it’s quite a chore for me to get up and get ready to go to the doctor.
[Over the phone is] easier because I don’t have to look at nobody and talk. (Laughs)
I told [my provider] about my pain and she made sure I had all the medications that I needed at the pharmacy. And she asked me what I needed and she made sure that that was there. So I was grateful for that because I was out of pain meds
**Providers: ease of access, improved monitoring, and urgent care availability**
You know [patients] pick up their phones. I guess it’s a convenience factor, it’s much easier to pick up your phone than actually travel to the clinic. [And] it might have also been that a lot of people were unable to work for a period so they were available to us…[T]he vast majority, I’d say 90% of the care is happening through phone right now and that’s been…good in some ways. Our show rates are a lot better.
I think there is also a big group of people that you know were uncontrolled [for chronic health conditions] because they would never come in and see us that now we’ve been able to track [them] down by phone…And our pharmacist at [this clinic] does a lot of chronic care management for diabetes and hypertension so have done a very good job with outreach for many of the patients they were following…But yeah that cohort of people who we just could never find now we’ve found some of them and cured them.

### Challenges of Telehealth

Both providers and patients identified challenges associated with the transition to telehealth. These challenges focused on the mechanics of telehealth access and delivery, including discomfort or unfamiliarity with and lack of access/low digital literacy of telehealth modalities; provider-patient communication during telehealth visits; privacy concerns; and provider decision-making about the necessity of in-person urgent care visits for medically complex patients.

#### Access to and use of telehealth technology

A small number of patients identified concerns about “being lost in the system” when telehealth was first initiated. These patients experienced challenges accessing providers and appointment scheduling. Some reported missing telehealth visits, because they did not recognize the new format as equivalent to a clinical visit. Providers expressed concerns about patients being mostly older adults, who were accustomed to in-person care in clinical settings. Patients echoed these concerns, some of whom initially rejected telehealth completely, identifying it as a care modality that would not work for them, even on a temporary basis. One patient stated: “I told [my primary care provider] until this [COVID-19 is] over there’s not really any point in meeting because I want to see you, I don’t want to hear you.”

#### Communication challenges during telehealth

The lack of a video format led to provider concerns about limited ability to diagnose and treat some conditions (e.g., rashes) and to assess patients’ pain without the ability to read facial expressions and body language during the telehealth interaction. Many patients reported increased pain severity resulting from shelter in place-related conditions. Phone-only visits meant providers lost important visual cues for pain assessment during a period when patients options for pain management (e.g., exercise, acupuncture, physical therapy) were curtailed or discontinued. Patients’ limited internet access and digital literacy inhibited clinics from initiating video-enabled visits. One provider reported: “It’s not the same where you’re there, face-to-face with somebody and they’re able to see your reaction, you see their reaction and you’re able to make sure they understand. I mean it’s good that I’m doing it over the phone but it’s not the same for me.”

Providers expressed concerns that telehealth may contribute to limitations in symptom assessment because the format does not lend itself well to longer conversations that inventory a range of potential health issues and multiple symptoms, as an in-person visit. Patients echoed this concern, indicating that they sometimes struggled to describe health issues over the phone. One patient remarked: “[The clinic’s] only calling you back based on immediate need of like urgent situations like physical situations so like symptoms. So that’s not yeah there is no – anything that has to do with [mental health] is not getting prioritized right now.”

#### Single-issue discussions and privacy concerns

Although both patients and providers identified telehealth as convenient and efficient, they noted that telehealth visits tended to focus discussions on only one problem. Providers preferred telehealth for brief visits. Patients’ concerns about privacy sometimes shortened telehealth visits. Housing situations sometimes constrained patients’ ability to freely share confidential information with their providers during telehealth visits. One patient stated: “I’m being as open as I can…with [my provider]…But at the time I was called [for a telehealth visit] I was sitting on the front porch and I’m thinking about everybody in the building is listening to me, which they can and they have, that’s been proven in the past. So I know that they can hear. And you know I’m very hard of hearing so I talk loudly. And so there’s no privacy there either.” 

#### Medical triage challenges with patients with complex chronic health conditions

Providers described the challenge of determining when patients needed to be prioritized for in-person visits, weighing the risks of patients with multiple chronic conditions traveling to the clinic verses the risks of their chronic conditions becoming poorly managed, necessitating hospitalization. One provider stated: Provider: “[T]he most vulnerable to COVID are people that you really often love to see [in clinic, in person] because you get their history and you also get the vitals and their physical. That’s the hardest thing for me. How to keep the sickest people safe [from COVID-19] but then out of the hospital. But then after the first month a lot of my really sick patients were really decompensating and ending up in the hospital.”

Patients sometimes disagreed with their providers about the urgency of an in-person visit. Patients who had planned procedures or testing postponed during the initial shelter-in place period reported feeling left in “limbo,” trapped between phone visits and follow up care. Some patients reported disengaging from care completely as a result of these challenges. One patient commented: “I totally lost contact with [my provider]. I don’t even know if she’s at [the clinic] anymore” Table 2.

**Table 2:** Challenges of telehealth

**Loss of connection between patients and providers**
[I am] definitely [concerned about] our patients who are unhoused. Before they would be patients that might be more likely to just drop into a clinic if they needed something or needed to make an appointment because they knew they could just do that. But now our building’s closed so they can’t. [Provider]
**Communication challenges during telehealth visits**
I mean the downside is people sometimes – there are people who can’t communicate effectively over the phone with you or they sometimes need you to help kind of guide them back to where the story goes. And I find those people to be very difficult to do on the phone. I think sometimes it’s hard to know whether people are minimizing their symptoms and you’re not looking at them so it’s kind of hard to tell over the phone. [Provider]
**Telephone format can over focus the clinical interaction**
When they’re in the room and they’re sitting down and you want to go through, “let’s go through all of your problems and all your healthcare maintenance and everything.” And I find now on the phone that really we’re focusing more on the one reason they called as opposed to all of the rest of the things that probably need to get done so I feel like we may be deferring care. [Provider]
**The digital divide**
I think for our patients there is also a pretty big digital divide so even if things that could be done maybe like video and show people how to do like their own things [e.g., blood pressure monitoring, etc.] […] I think that would be very hard to do just over the phone. [One of our clinic providers] tried…to pilot a Zoom™ call with one of his like 30-year-old, tech savvy patients. And [the patient] couldn’t do it. [Provider]
**Difficulty with diagnoses and decision-making about triage to in-person visit**
I had a 28-year-old young man with abdominal pain…And he was lower abdominal pain. But when you talked to them right on the phone I have a really hard time in sort of my training and my mindset is that the whole pain is really, really difficult to diagnose over the phone without actually touching somebody…And I don’t feel comfortable without like touching them. [Provider]

## Discussion

Patients and providers described increased convenience and decreased patient burden (e.g., travel, inconvenience) associated with telehealth. Opioid risk management strategies commonly used in this patient population include frequent check-in visits, requiring a significant time investment that was burdensome to both patients and providers [23]. Providers described benefits of telehealth in decreasing in-person visit frequency and travel burden on patients and to open space in busy clinician schedules. These benefits may be offset by risks of uncontrolled pain, increased substance use, and potential opioid safety and misuse concerns [16, 24]. During the COVID-19 pandemic, there have been substantial increases in drug overdose deaths across the US [25, 26]. However, the number of safety net patients with chronic non-cancer pain included in these statistics is currently unknown. Patients and providers in this study attributed increased uncontrolled pain and substance use to shelter-in-place limitations; increased isolation, anxiety, and depression associated with COVID-19; and patients’ disconnection from pain treatments and care. More research is needed to understand the risks and benefits of decreasing the burden of care access while also addressing the needs of medically complex patients with co-occurring opioid use disorder, chronic non-cancer pain, and multiple morbidities.

Our previous work documented that providers and patients can have divergent understandings about the risks associated with chronic opioid therapy [27]. In this study, we found providers and patients were largely in agreement about the benefits and challenges of telehealth. The shift to telehealth may present an opportunity to prioritize flexible patient-centered models, rather than patient monitoring focused primarily on medication adherence [28]. Research indicates that among patients with opioid use disorder, use of telehealth can increase patient engagement over time, yet telehealth’s role in expanding access to care, improving medication recall, and increasing patients’ level of attendance is not well understood, especially in urban settings [13, 14]. Providers in our study reported that telehealth enabled beneficial insights into patient’s home environments and family situations that informed clinical interactions. Telehealth’s potential role in increasing continuity of care from clinical to home settings is underexplored among patients with co-occurring opioid use disorder and chronic non-cancer pain, including the potential expansion of integrated pain management programs that offer non-pharmacologic pain treatments [29].

Telehealth video visits are associated with increased patient understanding and satisfaction compared with telephone-only communication [30]. One study found no significant differences in additional substance use, time to abstinence, or treatment retention between video-enabled telepsychiatry and face-to-face visits for opioid use disorder patients treated with buprenorphine [31]. Use of video may be critically important for patients with co-occurring opioid use disorder and chronic non-cancer pain who seek care in primary care settings, as ongoing relationships and clear communication are an integral part of chronic disease management [32, 33]. Current patient-facing telehealth software applications are not designed for patients with low-health literacy, making effective utilization difficult for patients with limited digital literacy, health literacy, and English proficiency [30]. Given the rapid rollout of telehealth during COVID-19 pandemic, it is important to monitor its use and the ways it exacerbates or mitigates existing vulnerabilities and disparities [34]. The worsening opioid overdose crisis and unclear resolution of the COVID-19 pandemic in the US necessitates urgency in addressing the challenges of digital inequity through practice and research. Clinicians will need training on the integration of telehealth modalities to complement and enhance existing competencies for the care of patients co-occurring opioid use disorder and chronic non-cancer pain [35].

This study has limitations. While we documented a range of patient experiences with telehealth implementation, patients who experienced long-term care disruptions during the COVID-19 pandemic are not included in this analysis, due to lack of longitudinal follow-up. While the range of clinics, patients and clinicians provide a breath of experiences, our sample size is relatively small (7 clinicians and 22 patients). Only established patients were enrolled in this study; it is possible that their perceptions about telehealth may be different from newer patients. As is common in qualitative studies, results may not be generalizable to a wider patient population, particularly those not in safety net settings. Clinics sampled in this study did not have regular video visits, so we were not able to assess how different forms of telehealth (e.g., video vs. phone-only) impacted patient care.

Chronic non-cancer pain and opioid use disorder are highly prevalent conditions [4–6]. The frequent co-occurrence of chronic non-cancer pain and opioid use disorder with additional morbidities underscores a need for a healthcare systems response that decreases patient burden while increasing accessibility and convenience. At the same time, the US opioid overdose crisis continues to generate unprecedented excess mortality, making safe and effective management of co-occurring chronic non-cancer pain and opioid use disorder a critical public health priority [1–3]. There is a need to better understand of impacts telehealth on opioid safety, misuse, and to improve pain control through multi-modal treatment strategies [5, 29]. Urban, safety net primary care settings are under-researched healthcare delivery systems for telehealth that present opportunities, as well as significant equity challenges, as barriers might hinder access to telehealth, particularly impacting low-income patients [30]. In the future, safety net clinics may be able to leverage telehealth to increase provider access and availability and develop flexible patient-centered models for patients with complex health needs. This should include the use of translation services during telehealth appointments to assess whether the language barrier is exacerbated by virtual appointment technology between providers and patients who do not speak the same language.

## Conclusion

While the COVID-19 pandemic accelerated adoption of telehealth primary care, it is likely to continue, in some form, into the foreseeable future [13]. This study is one of the first to examine the benefits and challenges of telehealth in a patient population with co-occurring substance use, chronic non-cancer pain, and multiple morbidities. The post-COVID-19 era will necessitate decisions about the continuation or expansion of telehealth for patients with opioid use disorder, chronic non-cancer pain, and multiple morbidities that receive care in urban, safety net settings by federal and state governmental bodies [11]. Our findings suggest that future research and training priority areas include: improving risk assessment tools for providers treating patient with co-occurring opioid use disorder and chronic non-cancer pain via telehealth; addressing the digital divide; and, expanding non-pharmacologic, home-based pain management strategies.

## Data Availability

The datasets generated and analyzed during the current study are not publicly available because it is not possible to effectively protect participant confidentiality in verbatim, qualitative transcript data.

## Abbreviations

SARS-CoV-2: COVID-19
ECROP: Examining the Consequences of Reductions in Opioid Prescribing
IRB: Institutional Review Board
PDMP: Prescription Drug Monitoring Program
SF: San Francisco
UCSF: University of California, San Francisco
US: United States
